# Supplementary material for: Mycolactone causes destructive Sec61-dependent loss of the endothelial glycocalyx and vessel basement membrane: a new indirect mechanism driving tissue necrosis in Mycobacterium ulcerans infection
Source: bioRxiv. 2024 Oct 1:2023.02.21.529382. Originally published 2023 Feb 21. Preprint. [Version 2] doi: 10.1101/2023.02.21.529382 (PMC9980099; doi:10.1101/2023.02.21.529382)
Supplement: Supplement 1 [file NIHPP2023.02.21.529382v2-supplement-1.pdf]

bioRxiv preprint doi: https://doi.org/10.1101/2023.02.21.529382; this version posted October 1, 2024. The copyright holder for this preprint (which was not certified by peer review) is the author/funder, who has granted bioRxiv a license to display the preprint in perpetuity. It is made available under aCC-BY 4.0 International license.

Supplementary Figures

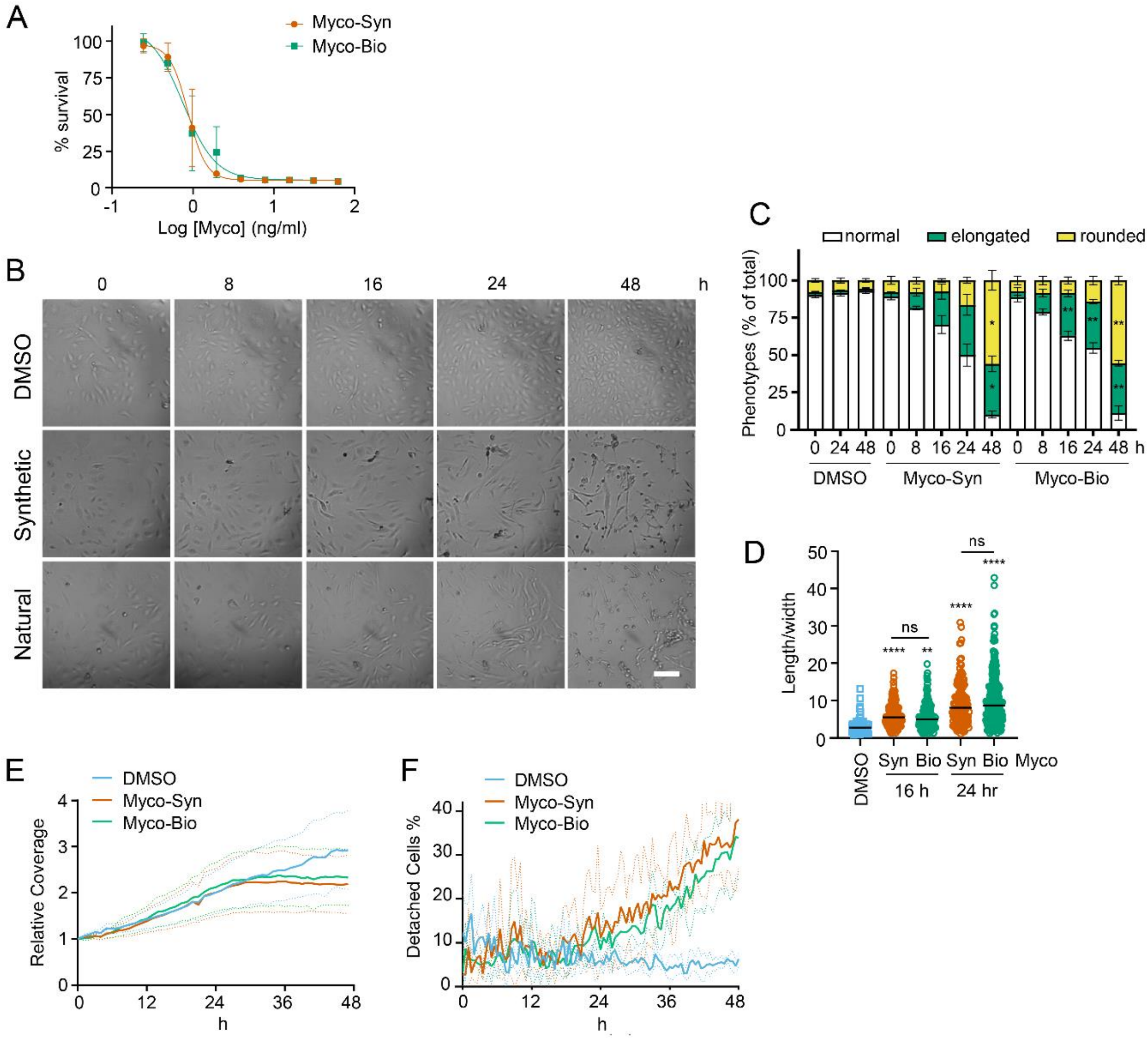

Figure S1. Sec61 inhibition alters endothelial cell morphology. **A.** Dose response of HDMEC to mycolactone produced synthetically (Myco-Syn) or purified from natural sources (Myco-Bio). Cells were exposed in triplicate to serial dilutions of compound for 96hr and viability measured with resazurin. Results are expressed as a percentage of the DMSO control. Data represents the mean of 3 independent experiments ± SEM. **B**. Cells treated with 10ng/ml synthetic (Syn) or natural (Nat) or 0.02% DMSO were imaged using the ZenCELL owl at indicated times. Scale bar =50μ. Images are representative of three independent experiments **C**.Cell numbers of each phenotype were counted and presented as a percentage of total cell number per field in (**B**) (mean ± SD for triplicate wells) *, *p*<0.05; **,*p*<0.01, n=1. **D.** Length and width of cells exposed to mycolactone for 16 and 24 hours or DMSO for 24 hours measured and presented as a ratio. **,*p*<0.01; ****, *p*<0.0001, Data is representative of duplicate independent experiments. **E-F.** Analysis determined by zenCell Owl inbuilt algorithms for images collected over 48hrs of cell coverage (**E**) and detached cells (**F**). Data represents the mean ± SD for triplicate wells, Data is representative of duplicate independent experiments.

bioRxiv preprint doi: https://doi.org/10.1101/2023.02.21.529382; this version posted October 1, 2024. The copyright holder for this preprint (which was not certified by peer review) is the author/funder, who has granted bioRxiv a license to display the preprint in perpetuity. It is made available under aCC-BY 4.0 International license.

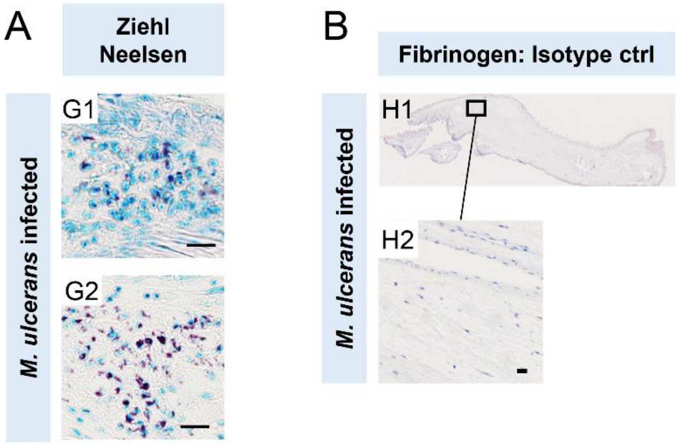

Figure S2. Profile of Buruli ulcer mouse footpad model. C57BL/6 mice receiving $1*10^5$ colony forming units *M. ulcerans* Mu_1082 strain injected intradermally into the footpad were sacrificed at 21or 28 days. Injected feet were fixed, decalcified and embedded in paraffin. (**A**) Sections stained for acid fast bacilli with Ziehl Neelson stain from mice infected for 21 days (A1) or 28 days (A2). At 21 days immune cell infiltration as well as apparently intracellular bacteria can be clearly seen in proximity to mycobacterial clusters (**B**) *M. ulcerans* mouse feet infected stained with isotype control antibody. Scale bars: 20 µm.

bioRxiv preprint doi: https://doi.org/10.1101/2023.02.21.529382; this version posted October 1, 2024. The copyright holder for this preprint (which was not certified by peer review) is the author/funder, who has granted bioRxiv a license to display the preprint in perpetuity. It is made available under aCC-BY 4.0 International license.

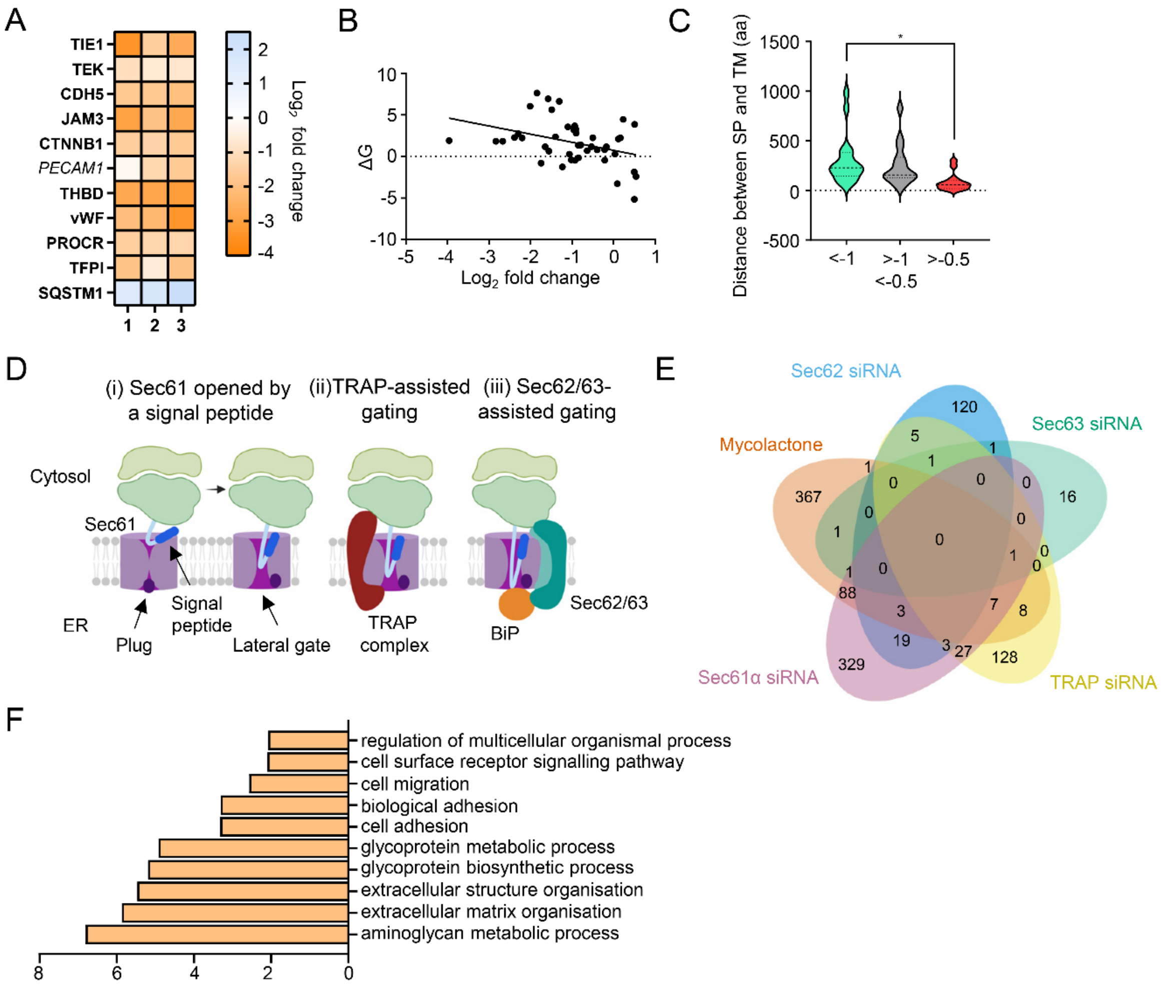

Figure S3. Proteomic analysis reveals loss of proteins associated with glycosylation, adhesion and migration. (**A**) Heat map showing fold change detected in this dataset for previously validated endothelial cell mycolactone targets (Ogbechi et al, 2015, Hsieh et al 2022). Dual-colour coding is shown., significantly downregulated ($p < 0.05$) or not ($p \geq 0.05$) in bold or *Italic* respectively. (**B**) Significant association between multipass protein membrane protein signal peptide (SP) ΔG values and level of downregulation by mycolactone ($p<0.05$). Membrane protein type and SP sequence based on Uniprot data, ΔG quantified using ΔG Prediction Server V1.0 (https://dgpred.cbr.su.se). (**C**). Impact of distance between signal peptide and first transmembrane domain on susceptibility of multipass proteins to downregulation by mycolactone. Statistical analysis was performed by one-way ANOVA with Tukey's correction for multiple comparisons in GraphPad Prism Version 9.4.1. *, $p<0.05$ (**D**) Model depicting assisted and unassisted channel opening by signal peptides. (i) Binding of a nascent chain signal peptide to Sec61α opens a lateral gate into the membrane and causes a shift in the position of the plug domain, allowing access to the ER lumen. (ii) TRAP increases translocation of proteins whose signal peptides bear a high GP content. TRAP is a heterotetrameric complex which interacts with the ribosome on the cytosolic side of the ER membrane and with Sec61α on the luminal side, binding to a hinge region between the N- and C-terminal halves of the protein facilitating channel opening [1, 2] (iii) The Sec62/63 complex is involved in post-translational translocation but can also assist opening of the channel for proteins with signal peptides that gate slowly due to the presence relatively long but less hydrophobic "H-regions" and lower carboxy terminal polarity [3]. This complex also interacts with the ribosome and the translocon and may also interact directly with the nascent peptide chain [4]. In addition, Sec63 recruits BiP to the translocon to further assist channel opening on the luminal side [3]. (**E**) Overlap between mycolactone-downregulated endothelial membrane proteome and translocon-dependent proteome. Venn diagram created using JVenn [5] showing overlap in significantly downregulated proteome between the dataset presented here and those obtained in Hela cells treated with siRNA for Sec61α and translocon associated protein TRAP or HEK293 cells with Sec62 or Sec623 knocked out [1, 3]. (**F**) Top significantly over-represented ($p<0.05$) Gene Ontology groups in upregulated data set, compared to whole genome. Data generated with WebGestalt.

bioRxiv preprint doi: https://doi.org/10.1101/2023.02.21.529382; this version posted October 1, 2024. The copyright holder for this preprint (which was not certified by peer review) is the author/funder, who has granted bioRxiv a license to display the preprint in perpetuity. It is made available under aCC-BY 4.0 International license.

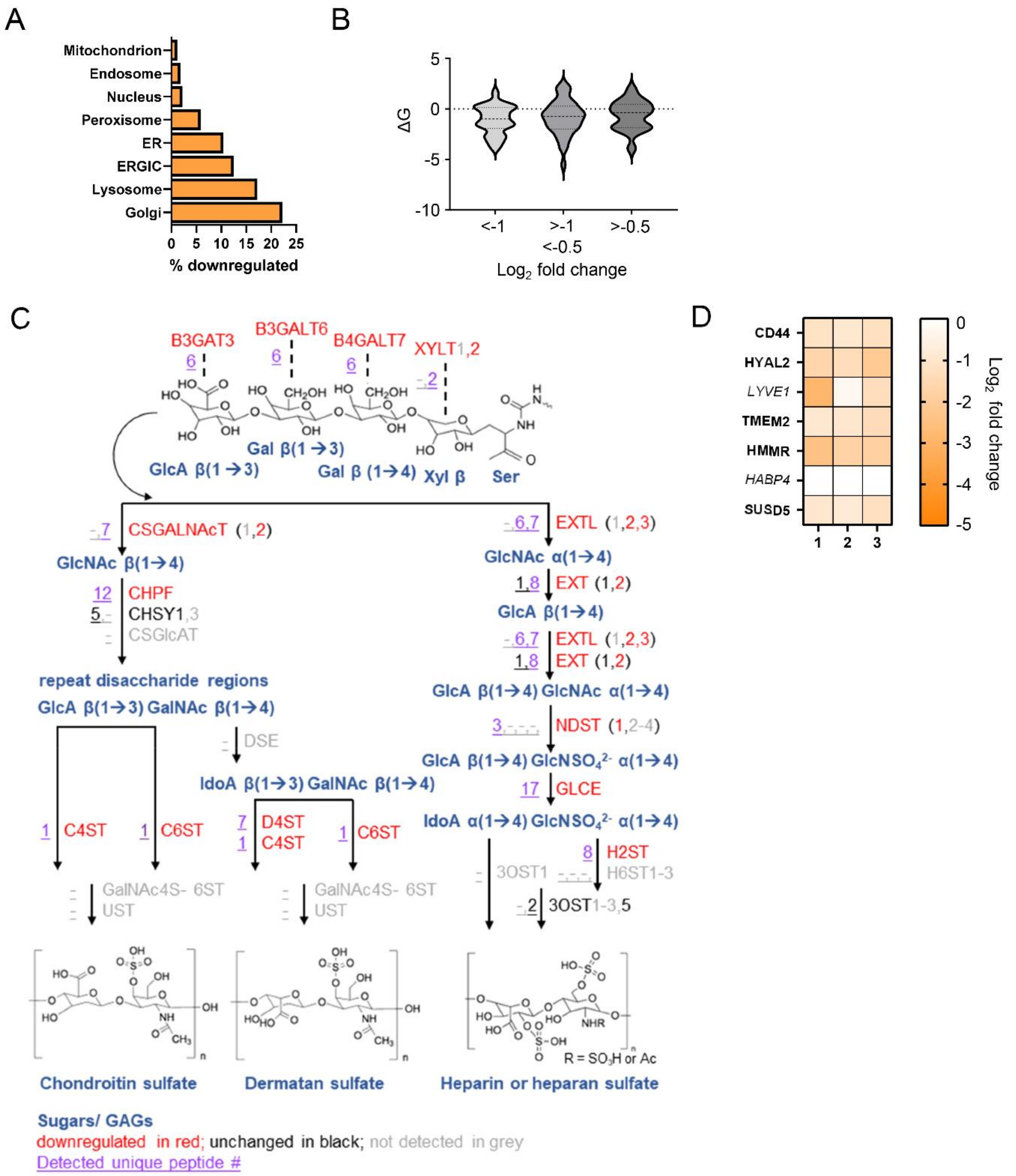

Figure S4. Mycolactone targets Golgi proteins involved in glycosaminoglycan chain synthesis initiation.. **(A)** Downregulated intracellular proteins in HDMECs following 24 hours exposure to 10 ng/mL mycolactone, according to subcellular location, presented as percentage of total. **(B)** Impact of membrane anchor ΔG values on fold change in expression induced by mycolactone for all identified Type II membrane proteins. (**C**) Cartoon representing mycolactone-downregulated steps of GAG enzymatic synthesis. Each intermediate product is shown in blue and alongside with its responsible enzyme(s). Candidates significantly downregulated ($p < 0.05$) by mycolactone are shown in red, unchanged in black and undetected in grey. Unique peptide numbers detected in proteome are in purple (underlined).

bioRxiv preprint doi: https://doi.org/10.1101/2023.02.21.529382; this version posted October 1, 2024. The copyright holder for this preprint (which was not certified by peer review) is the author/funder, who has granted bioRxiv a license to display the preprint in perpetuity. It is made available under aCC-BY 4.0 International license.

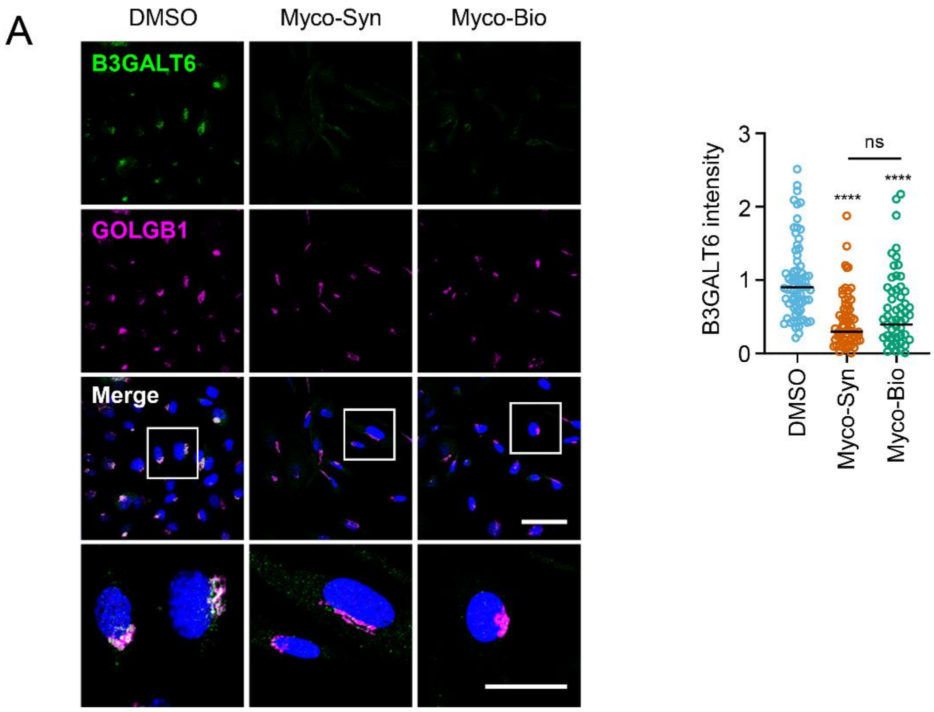

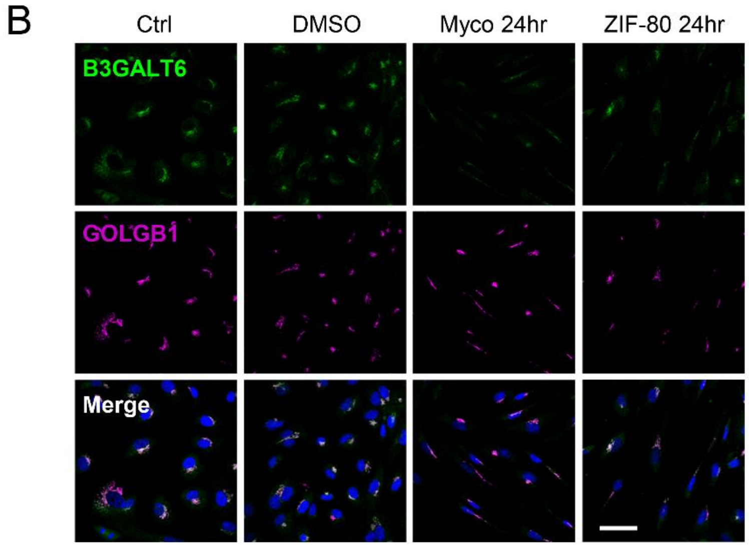

Figure S5. Sec61 blockade suppresses B3GALT6 expression in endothelial cells. A. HDMECs exposed to 10 ng/mL synthetic (Myco-Syn) or natural mycolactone (Myco-Bio) or 0.02% DMSO for 24hr were fixed, permeabilised and immunostained with anti-B3GALT6 and anti-giantin antibodies. **B3GALT6** (green) and the Golgi apparatus (magenta) were visualised and nuclei stained with DAPI (blue). Scale bar = 50 μm (20 μm in the crop panels). Corrected total cell fluorescence of B3GALT6 in Golgi apparatus per cell measured and presented as a value normalised to the mean value obtained from untreated control of each experiment. At least 60 cells per condition were measured. Data is representative of duplicate experiments. ****, *p*<0.0001. (**B**) HDMECs exposed to 10 ng/mL of mycolactone (Myco), 0.02% DMSO, 20 nM ZIF-80 or untreated for 24 hours were fixed, permeabilised and immunostained with anti-B3Galt6 and anti-giantin antibodies and nuclei stained with DAPI. Images are representative of 2 independent experiments. Scale bar = 50 μm. Statistical analysis was performed by one-way ANOVA with Dunnett's correction for multiple comparisons in GraphPad Prism Version 9.4.1

bioRxiv preprint doi: https://doi.org/10.1101/2023.02.21.529382; this version posted October 1, 2024. The copyright holder for this preprint (which was not certified by peer review) is the author/funder, who has granted bioRxiv a license to display the preprint in perpetuity. It is made available under aCC-BY 4.0 International license.

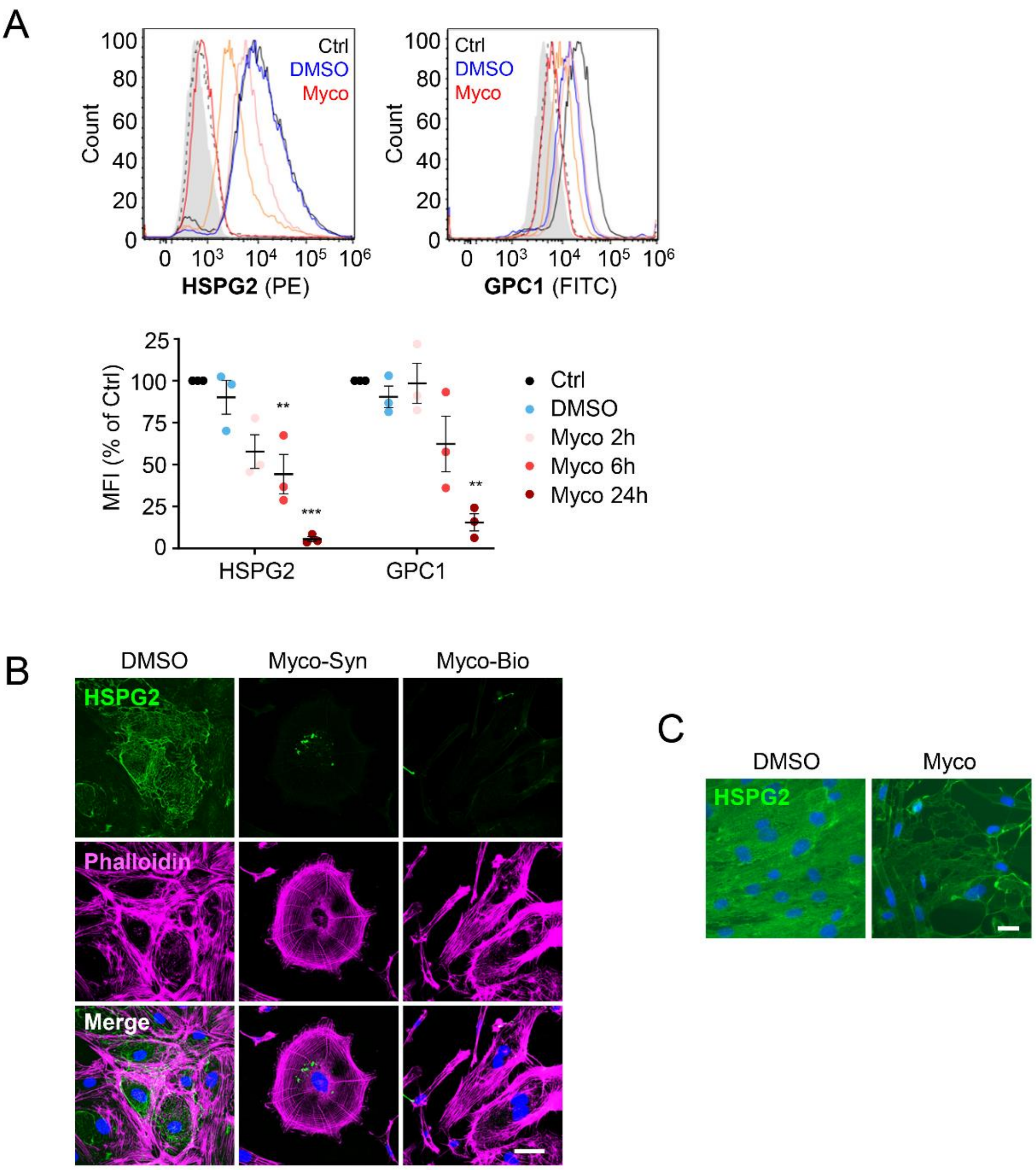

Figure S6. Mycolactone's effect on endothelial surface proteoglycans. HUVECs exposed to 10 ng/mL of mycolactone (Myco), 0.02% DMSO or remained untreated for indicated times. Cells were harvested for flow cytometry analysis. Histogram plots for single cell population of perlecan and glypican-1. Unstained, untreated cells, filled grey; isotype control of untreated cells, dashed black line. untreated cells stained with antibodies, black line; cells exposed to DMSO stained with antibodies, blue line; cells exposed to mycolactone for 2, 6, and 24 hours stained with antibodies, pink, orange and red line, respectively. MFI is presented as a % of untreated control (mean ± SEM of 3 independent experiments). **B**. HDMECs exposed to 10 ng/mL synthetic (Syn) or natural mycolactone (Nat) or 0.02% DMSO for 24 hours. Cells were fixed and immunostained with anti-perlecan antibody (green), permeabilised and labelled with TRITC-conjugated phalloidin (magenta). Nuclei were stained with DAPI (blue). Scale bar = 20 μm, n=1. **C.** Confluent HDMEC incubated on a rotary shaker for 24hr before addition of 0.02% DMSO or 10ng/ml mycolactone (Myco) for 48hr were fixed and stained with anti-perlecan antibody (green) and counterstained with DAPI (blue). Scale bar = 10 μm, n=1.. Statistical analysis was performed by one-way ANOVA with Dunnett's correction for multiple comparisons in GraphPad Prism Version 9.4.1. ns, not significant; **, $P < 0.01$; ***, $P < 0.001$.

bioRxiv preprint doi: https://doi.org/10.1101/2023.02.21.529382; this version posted October 1, 2024. The copyright holder for this preprint (which was not certified by peer review) is the author/funder, who has granted bioRxiv a license to display the preprint in perpetuity. It is made available under aCC-BY 4.0 International license.

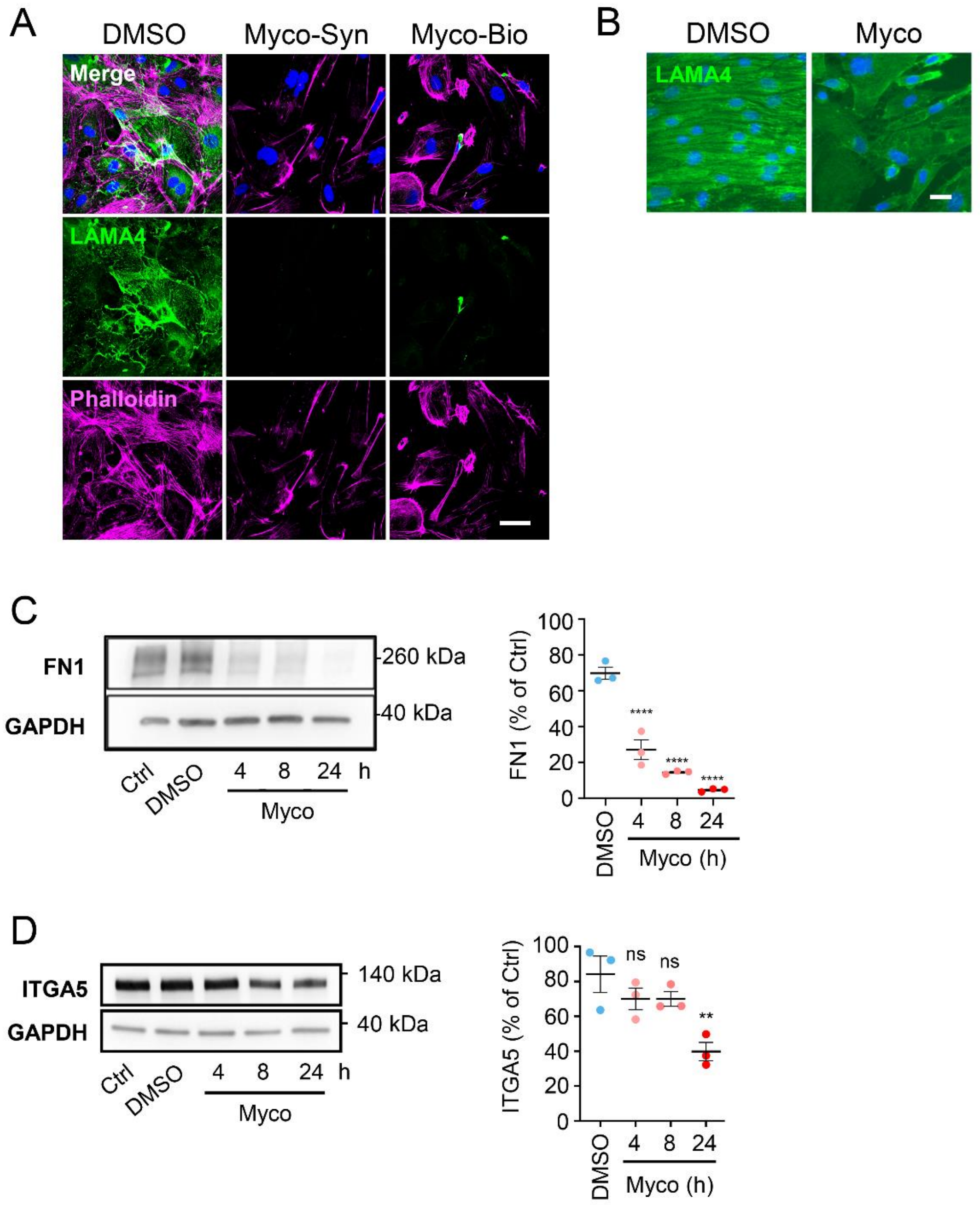

Figure S7. Mycolactone impacts endothelial cell adhesion molecules. A. HDMECs exposed to 10 ng/mL synthetic (Myco-Syn) or natural mycolactone (Myco-Bio) or 0.02% DMSO for 24 hours. Cells were fixed and immunostained with anti-LAMA4 antibody (green), permeabilised and labelled with TRITC-conjugated phalloidin (magenta). Nuclei were stained with DAPI (blue). Scale bar = 20 µm, n=1. **B**. Confluent HDMEC incubated on a rotary shaker for 24hr before addition of 0.02% DMSO or 10ng/ml mycolactone (Myco) for 48hr were fixed and stained with anti-LAMA4 antibody (green) and counterstained with DAPI (blue). Scale bar = 10 µm, n=1. **C-D**. HDMECs exposed to 10 ng/mL of mycolactone (Myco), 0.02% DMSO or remained untreated for indicated times. Cells were lysed and subjected to immunoblotting with anti-fibronectin (**C**) and anti-integrin α5 D) antibodies. Each immunoblot intensity was normalised according to GAPDH and untreated controls. Statistical analysis was performed by one-way ANOVA with Dunnett's correction for multiple comparisons in GraphPad Prism Version 9.4.1Data from 3 independent experiments are presented (mean ± SEM). ns, not significant; **, *P* < 0.01; ****, *P* < 0.0001.

bioRxiv preprint doi: https://doi.org/10.1101/2023.02.21.529382; this version posted October 1, 2024. The copyright holder for this preprint (which was not certified by peer review) is the author/funder, who has granted bioRxiv a license to display the preprint in perpetuity. It is made available under aCC-BY 4.0 International license.

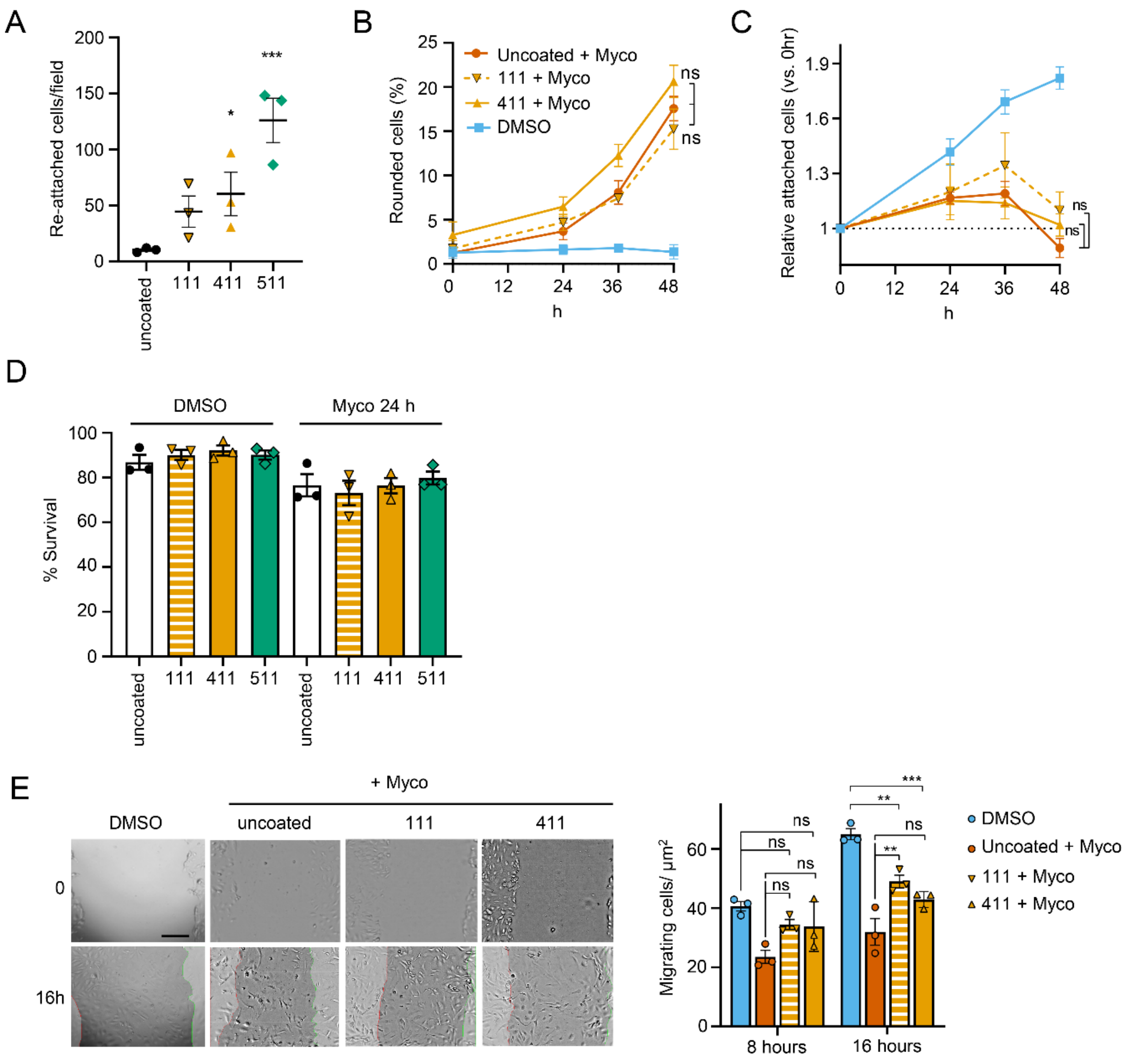

Figure S8. Endothelial cell adhesion to various laminin isoforms. (A) HDMECs were harvested and layered to laminin-511, 411, 111 or uncoated wells for one hour. Non-adherent cells were washed away and attached cells per field were counted. mean ± SEM (n = 3). **(B)** HDMECs seeded onto different laminin isoforms were untreated or exposed to 0.02% DMSO or 10 ng/mL mycolactone (Myco) for 48 hrs. Their viability was assayed using CellEvent detection kit as described in [6]. The number of live cells (negative for both active caspase 3/7 and PI) in three fields was determined and expressed as a proportion of total cells (Mean ± SEM, $n = 3$ independent experiments). Statistical analysis was carried out using GraphPad Prism Version 9.4.1. Panels **A&D** used a repeated measures one-way ANOVA with Dunnett's correction for multiple comparisons (**D** also included the Geisser Greenhouse correction for sphericity). Panels **B**,**C&E** used a two-way ANOVA with Tukey's correction for multiple comparisons (panels B&C also included the Geisser Greenhouse correction for sphericity). ns, not significant; *, $P < 0.05$; **, $P < 0.01$; ***, $P < 0.001$.

bioRxiv preprint doi: https://doi.org/10.1101/2023.02.21.529382; this version posted October 1, 2024. The copyright holder for this preprint (which was not certified by peer review) is the author/funder, who has granted bioRxiv a license to display the preprint in perpetuity. It is made available under aCC-BY 4.0 International license.

References

1. Nguyen D, Stutz R, Schorr S, Lang S, Pfeffer S, Freeze HH, et al. Proteomics reveals signal peptide features determining the client specificity in human TRAP-dependent ER protein import. Nat Commun. 2018;9(1):3765. Epub 2018/09/16. doi: 10.1038/s41467-018-06188-z.
2. Pfeffer S, Dudek J, Schaffer M, Ng BG, Albert S, Plitzko JM, et al. Dissecting the molecular organization of the translocon-associated protein complex. Nat Commun. 2017;8:14516. Epub 2017/02/22. doi: 10.1038/ncomms14516.
3. Schorr S, Nguyen D, Hassdenteufel S, Nagaraj N, Cavalie A, Greiner M, et al. Identification of signal peptide features for substrate specificity in human Sec62/Sec63-dependent ER protein import. FEBS J. 2020;287(21):4612-40. Epub 2020/03/07. doi: 10.1111/febs.15274.
4. Conti BJ, Devaraneni PK, Yang Z, David LL, Skach WR. Cotranslational stabilization of Sec62/63 within the ER Sec61 translocon is controlled by distinct substrate-driven translocation events. Mol Cell. 2015;58(2):269-83. Epub 2015/03/25. doi: 10.1016/j.molcel.2015.02.018.
5. Bardou P, Mariette J, Escudie F, Djemiel C, Klopp C. jvenn: an interactive Venn diagram viewer. BMC Bioinformatics. 2014;15:293. Epub 2014/09/02. doi: 10.1186/1471-2105-15-293.
6. Ogbechi J, Hall BS, Sbarrato T, Taunton J, Willis AE, Wek RC, et al. Inhibition of Sec61-dependent translocation by mycolactone uncouples the integrated stress response from ER stress, driving cytotoxicity via translational activation of ATF4. Cell Death Dis. 2018;9(3):397. Epub 2018/03/16. doi: 10.1038/s41419-018-0427-y.
